# Disrupting glucose-6-phosphate isomerase fully suppresses the “Warburg effect” and activates OXPHOS with minimal impact on tumor growth except in hypoxia

**DOI:** 10.18632/oncotarget.21007

**Published:** 2017-09-18

**Authors:** Monique Cunha de Padua, Giulia Delodi, Milica Vučetić, Jérôme Durivault, Valérie Vial, Pascale Bayer, Guilhermina Rodrigues Noleto, Nathalie M. Mazure, Maša Ždralević, Jacques Pouysségur

**Affiliations:** ^1^ Université Côte d’Azur, IRCAN, CNRS, Inserm, Centre A Lacassagne, Nice, France; ^2^ Medical Biology Department, Centre Scientifique de Monaco (CSM), Monaco; ^3^ Université Côte d’Azur, University Hospital Pasteur, Clinical Chemistry Laboratory, Nice, France; ^4^ Department of Biochemistry and Molecular Biology, Federal University of Parana, Curitiba, Brazil

**Keywords:** glycolysis, OXPHOS, pentose phosphate pathway, glucose-6-phosphate isomerase, tumor growth

## Abstract

As Otto Warburg first observed, cancer cells largely favor fermentative glycolysis for growth even under aerobic conditions. This energy paradox also extends to rapidly growing normal cells indicating that glycolysis is optimal for fast growth and biomass production. Here we further explored this concept by genetic ablation of fermentative glycolysis in two fast growing cancer cell lines: human colon adenocarcinoma LS174T and B16 mouse melanoma. We disrupted the upstream glycolytic enzyme, glucose-6-phosphate isomerase (*GPI*), to allow cells to re-route glucose-6-phosphate flux into the pentose-phosphate branch. Indeed, *GPI*-KO severely reduced glucose consumption and suppressed lactic acid secretion, which reprogrammed these cells to rely on oxidative phosphorylation and mitochondrial ATP production to maintain viability. In contrast to previous pharmacological inhibition of glycolysis that suppressed tumor growth, *GPI*-KO surprisingly demonstrated only a moderate impact on normoxic cell growth. However, hypoxic (1% O_2_) cell growth was severely restricted. Despite *in vitro* growth restriction under hypoxia, tumor growth rates *in vivo* were reduced less than 2-fold for both *GPI*-KO cancer cell lines. Combined our results indicate that exclusive use of oxidative metabolism has the capacity to provide metabolic precursors for biomass synthesis and fast growth. This work and others clearly indicate that metabolic cancer cell plasticity poses a strong limitation to anticancer strategies.

## INTRODUCTION

In contrast to normally differentiated cells that derive their energy from oxidative phosphorylation (OXPHOS), it is now well documented that the majority of rapidly developing tumors depend primarily on fermentative glycolysis even when oxygen is plentiful, a phenomenon referred to as the “Warburg effect” [1–4]. This high glycolytic phenotype, also called ‘glycolytic addiction’, results from the conjunction of uncontrolled growth signaling, deregulated c-Myc and hypoxia-induced factor 1 (HIF-1) activity leading to induction of glycolytic enzymes and inhibition of mitochondrial pyruvate oxidation [5–7]. This unique metabolic cancer phenotype has prompted many studies to investigate whether specific inhibition of glycolysis in tumors may have therapeutic benefit. Initially, studies exploited inhibition of the first glycolytic step with 2-deoxy-glucose (2-DG), a competitive inhibitor of glucose transport but also an inhibitor of hexokinase and glucose-6-phosphate isomerase [4, 8, 9]. Unfortunately, clinical use of 2-DG is greatly limited due to toxicity as it inhibits the dual metabolic flux of glycolysis and oxidative Pentose Phosphate Pathway (PPP). Other investigators have explored inhibition, gene silencing or disruption of specific downstream steps of glycolysis, namely lactate dehydrogenases A and B [10–13] or the final step of lactic acid export *via* the H^+^/lactate symporters (monocarboxylate transporters 1 and 4, MCT1 and MCT4) [14–17].

Here we report on the metabolic adaptation and consequent impact on tumor growth following disruption of the most upstream glycolytic step, the inter-conversion between glucose-6-phosphate (G6P) and fructose-6-phosphate (F6P), catalyzed by Glucose-6-Phosphate Isomerase (GPI), with preservation of the oxidative PPP metabolic flux. Genetic disruption of *GPI* was generated in two aggressive cancer cell lines: the human colon adenocarcinoma LS174T and the mouse B16 melanoma. Both of these cell lines display a high glycolytic rate and rapid tumor growth in immune-compromised mice [11, 17]. As expected, *GPI*-knockout (*GPI*-KO) ablated the fermentative glycolytic flux in the two cell lines as measured by the full suppression of lactic acid release in normoxia or hypoxia (less than 1% of the parental cell value). Surprisingly, *GPI*-KO cell growth in normoxia is minimally reduced and maintained by a reprogrammed metabolic oxidative phenotype, essential for anabolism, ATP biosynthesis, viability and *in vitro* growth. Consequently, these *GPI*-KO mutant cancer cells became oxygen-dependent for growth and therefore extremely sensitive to respiratory chain inhibitors.

## RESULTS

### Genetic disruption of Glucose-6-phosphate isomerase (*GPI*) in LS174T and B16 cancer cell lines induces expression of thioredoxin interacting protein (TXNIP)

**Figure 1:**
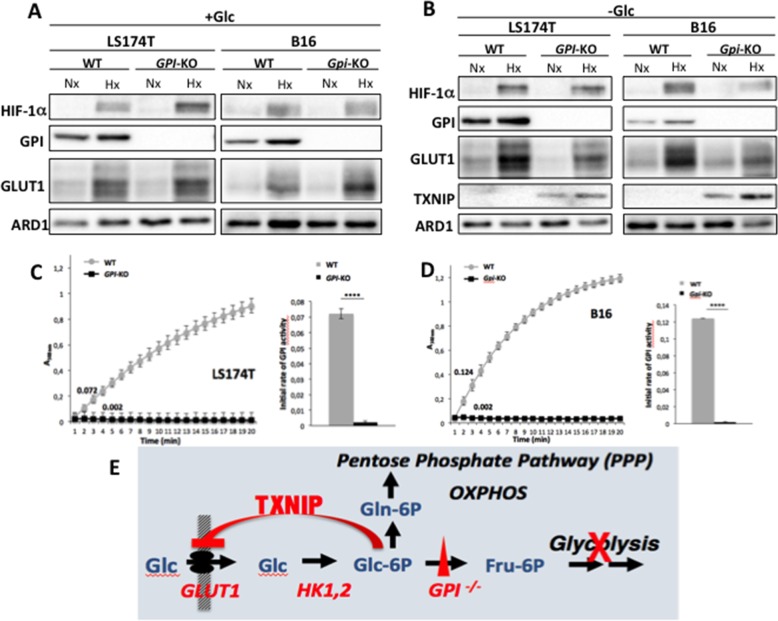
*GPI* disruption affects GLUT1 and TXNIP expression levels and GPI-enzymatic activities in LS174T and B16 cells Immunoblot analysis of HIF-1α, GPI, GLUT-1 and TXNIP after 24h in normoxia (Nx) and hypoxia (Hx), in WT and *GPI*-KO cells in the presence of 25 mM glucose (+Glc) **(A)** or absence of glucose (-Glc) **(B)**. ARD1 was used as loading control. **(C, D)** enzymatic assay of GPI activity in WT and *GPI*-KO cells was performed by continuous spectrophotometric rate determination. Numbers along the curves represent initial rates of the reaction. The results presented are representative of four independent experiments. In the right inserts of both figures, average value of the initial rates of GPI activity in WT and *GPI*-KO cells was plotted. ^****^ p < 0.0001. **(E)** Schematic representation of the regulation of glucose transport in *GPI*-KO cells.

Previous attempts to knock-down *GPI* in the LS174T cancer cell line with doxycycline-inducible short hairpin (sh) RNA showed that 90% silencing of GPI enzymatic activity did not change the rate of glycolysis, indicating the limits of shRNA use for non-limiting enzymes (Laferrière J. and Pouyssegur J. unpublished results). We therefore disrupted the *GPI* gene with CRISPR/Cas9 in both human colon adenocarcinoma LS174T and mouse B16 melanoma cell lines (Figure 1). The complete disruption of *GPI* alleles was attested by immunoblotting in normoxia (Nx) and hypoxia (Hx) (Figures 1A and 1B) and more importantly by enzymatic assay (Figures 1C and 1D). The rate of GPI enzymatic activity was monitored spectrophotometrically, as described in the Materials and Methods section, and was shown to be completely abolished in the two *GPI*-KO cell lines, confirming functional invalidation of the protein.

Interruption of the glucose flow at the GPI glycolytic step is assumed to increase intracellular glucose-6-phosphate (G6P). An interesting proposed nuclear sensor of this metabolite is the transcription factor complex, MondoA-Mlx, that induces among other genes Thioredoxin Interacting Protein (TXNIP), a potent negative regulator of glucose uptake [18]. Interestingly, this fine regulation by TXNIP triggers a feedback loop that restricts glucose transporter (GLUT1) activity [19], an “old” observation referred to as “hexose transport curb” [9, 20]. We indeed observed that in the conditions of 24h glucose starvation only the two cell lines disrupted for *GPI* expressed TXNIP in normoxia and hypoxia (Figure 1B), and had decreased GLUT1 expression. The exclusive expression of TXNIP in the two *GPI*-disrupted cell lines fully supports the fine-tuning feedback regulation of glucose transport [18, 19] illustrated in Figure 1E.

### Consequences of *GPI* disruption on *in vitro* growth in normoxia or hypoxia

**Figure 2:**
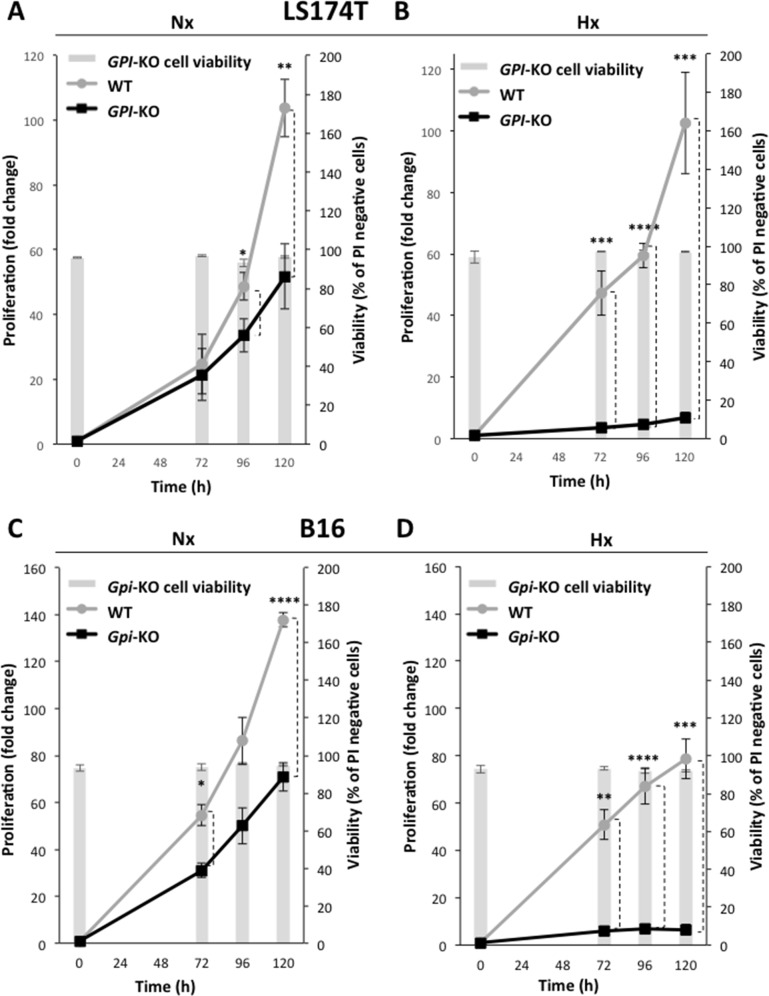
Cell growth and cell viability of WT and *GPI*-KO cells in normoxia and 1% hypoxia Cell proliferation and viability of LS174T WT and *GPI*-KO in Nx **(A)** or Hx **(B)** up to 120h. Cell proliferation of B16 WT and *Gpi*-KO in Nx **(C)** and in Hx **(D)**, up to 120h. The mean ± S.E.M. is representative of four independent experiments performed in duplicate. ^*^ p < 0.05, ^**^ p < 0.003, ^***^ p < 0.0003, ^****^ p < 0.0001.

We first analyzed and compared the exponential growth in regular medium (DMEM) of two independent *GPI*-KO clones issued from the highly glycolytic cell line LS174T [17]. Surprisingly, in Figure 2A, we show that the *in vitro* growth of *GPI*-KO cells in normoxia is reduced only about two-fold after 5 days. In contrast, the growth in hypoxia (1% O_2_) is severely restricted (> 90% inhibition) after 5 days (Figure 2B). Both in normoxia and hypoxia, cell viability remains unchanged (Figure 2A and 2B, bars, right scale), and cannot account for the reduced rate of cell proliferation. Identical findings were obtained for *GPI*-KO clone #2 (data not shown).

For the mouse melanoma cell line B16 that exhibits dual high rates of glycolysis and oxidative phosphorylation (OXPHOS) as seen later, *Gpi* disruption affected growth similarly as for the colon adenocarcinoma cell line LS174T. We observe about two-fold growth rate reduction in normoxia (Figure 2C) and almost total absence of growth in hypoxia (Figure 2D). Again, viability of B16 *Gpi*-KO was not affected after 5 days of culture neither in normoxia nor in hypoxia (Figure 2C, 2D, bars, right scale).

### *GPI*-knockouts fully suppress “Warburg effect” in colon and melanoma cancer cell lines

**Figure 3:**
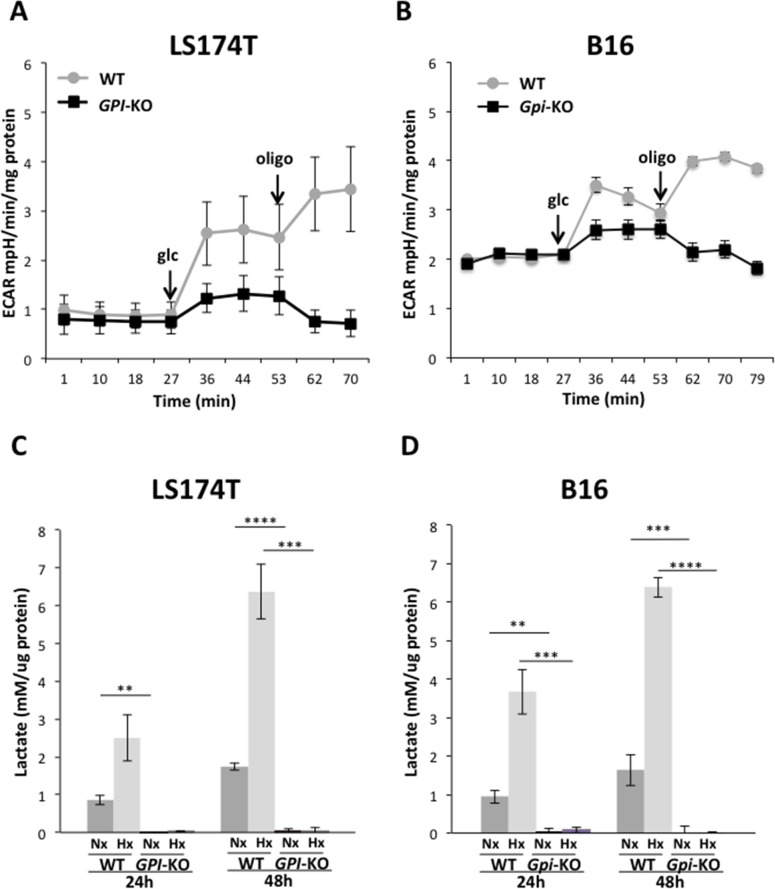
*GPI*-KOs disrupt glycolysis and abolish lactic acid production Extracellular acidification rate (ECAR) of LS174T **(A)** and B16 **(B)** WT and *GPI*-KO cells in Nx, analyzed with Seahorse XF24 bioanalyzer. The mean ± S.E.M. is representative of four independent experiments performed in quadruplicate. Lactate levels of WT and *GPI*-KO LS174T **(C)** and B16 **(D)** cells grown in Nx or 1% Hx secreted in 24- and 48-h and quantified by Cobass c701 (Roche). Data were normalized by protein content and the mean ± S.E.M. are representative of five independent experiments. ^**^ p < 0.01, ^***^ p < 0.005, ^****^ p < 0.0001.

The results of metabolic flux studies comparing wild type (WT) and *GPI*-KO in the two cancer lines are presented in Figure 3A and 3B. Upon glucose addition, a rapid and strong extracellular acidification rate (ECAR) is observed in both WT cell lines, and it is further increased (approximately doubled) upon oligomycin addition (Figures 3A and 3B), reflecting the cells glycolytic capacity. In contrast, the two *GPI*-KO cell lines display an identical profile: low acidic release upon glucose addition and absence of the acid release burst in response to oligomycin addition (Figures 3A and 3B). This very low ECAR profiles in *GPI*-KO cell lines are consistent with genetic interruption of the glycolytic flow.

Complete ablation of fermentative glycolysis in *GPI*-null cell lines is further demonstrated by measuring the release of lactic acid of cells cultivated under normoxic or hypoxic conditions (Figure 3C, 3D). Both WT cell lines are moderately glycolytic in normoxia, an action that is 6-fold stimulated when the cells were cultivated for 48h in 1% oxygen. Under the same conditions, *GPI*-null cells did not secrete lactic acid (no detectable in normoxia and less than 1% in hypoxia, Figure 3C, 3D) and glucose consumption was reduced by more than 95% compared to WT cell lines. Thus, the ECAR (acid release) observed in *GPI*-null cells in Figure 3A, 3B upon glucose addition must be due to carbonic and not lactic acid release, as we will confirm in the next section.

We conclude that *GPI* disruption fully eradicate fermentative glycolysis (“Warburg effect”) in the two cancer cell lines from colon adenocarcinoma and melanoma.

### *GPI*-knockouts re-direct glucose metabolism through OXPHOS in colon and melanoma cancer cell lines

**Figure 4:**
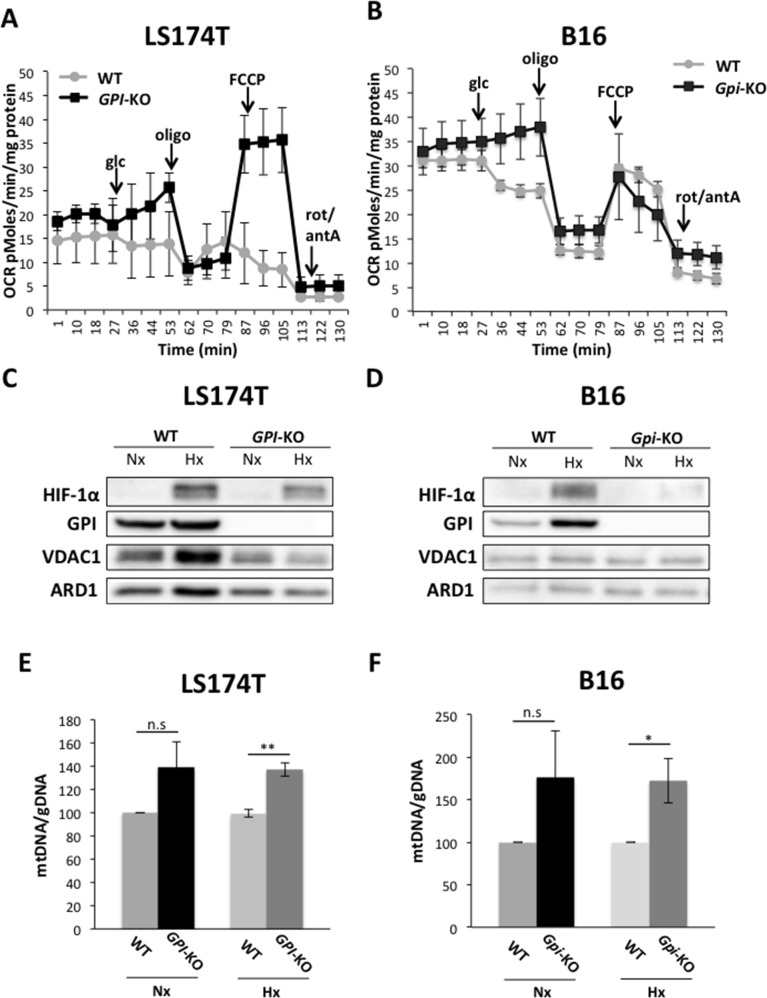
Oxidative metabolism of glucose is activated in *GPI*-KO cells Oxygen consumption rate (OCR) of LS174T **(A)** and B16 **(B)** WT and *GPI*-KO cells evaluated with Seahorse XF24 bioanalyzer. The mean ± S.E.M. is representative of four independent experiments performed in quadruplicate. VDAC1 protein levels in WT and *GPI*-KO cells in Nx and 1% Hx, in LS174T **(C)** and B16 **(D)** cells. **(E)** and **(F)** mtDNA/gDNA ratio content of WT and *GPI*-KO cells evaluated by qPCR. The mean ± S.E.M. is representative of four independent experiments performed in duplicate.^*^ p < 0.03, ^**^ p < 0.003.

Oxygen consumption rates (OCR), complementary of the experiment depicted in Figure 3A, 3B, are represented in Figure 4A, 4B. Addition of glucose in WT LS174T cells slightly decreases OCR and further additions of oligomycin and FCCP (carbonyl cyanide 4-(trifluoromethoxy) phenylhydrazone), a potent mitochondrial OXPHOS uncoupler, confirm that these glycolytic cells display a very low rate of oxygen consumption (Figure 4A), as previously reported [17]. In sharp contrast, addition of glucose to *GPI*-null LS174T cells (Figure 4A) causes a marked increase in OCR, abolished by oligomycin. Addition of FCCP, followed by Rotenone and Antimycin A indicates a very robust respiratory capacity on glucose *via* the Pentose Phosphate Pathway. The situation is different for the B16 WT (Figure 4B). This cell line, in spite of a very active glycolytic rate (Figure 3B, 3D), displays a rather strong respiratory capacity contrasting with that of LS174T WT cells. As it was the case with LS174T cell lines, B16 WT cells decrease their OCR and *Gpi*-null cells maintain the steady increased OCR upon glucose addition. This OCR is inhibited by oligomycin in both cell lines, reflecting the level of mitochondrial contribution to the ATP production, since oligomycin inhibits ATP synthase (Complex V) of the mitochondrial respiratory chain. However, the addition of uncoupler (FCCP) was unable to restore the respiration in *Gpi*-KO cells, indicating a possible mitochondrial dysfunction (Figure 4B).

The question of whether the abolition of fermentative glycolysis and re-activation of OXPHOS had increased mitochondrial content during the phase of mutant selection/expansion was addressed by assessing expression of the major mitochondrial channel VDAC1 (Figure 4C, 4D) and the mitochondrial (mt)/genomic (g) DNA ratio (Figure 4E, 4F). In both LS174T and B16 cells, *GPI*-KO had a slight increase in mt/gDNA ratio in hypoxia, but not higher VDAC1 expression with respect to the WT cells, thus this hypothesis has been rapidly eliminated.

**Figure 5:**
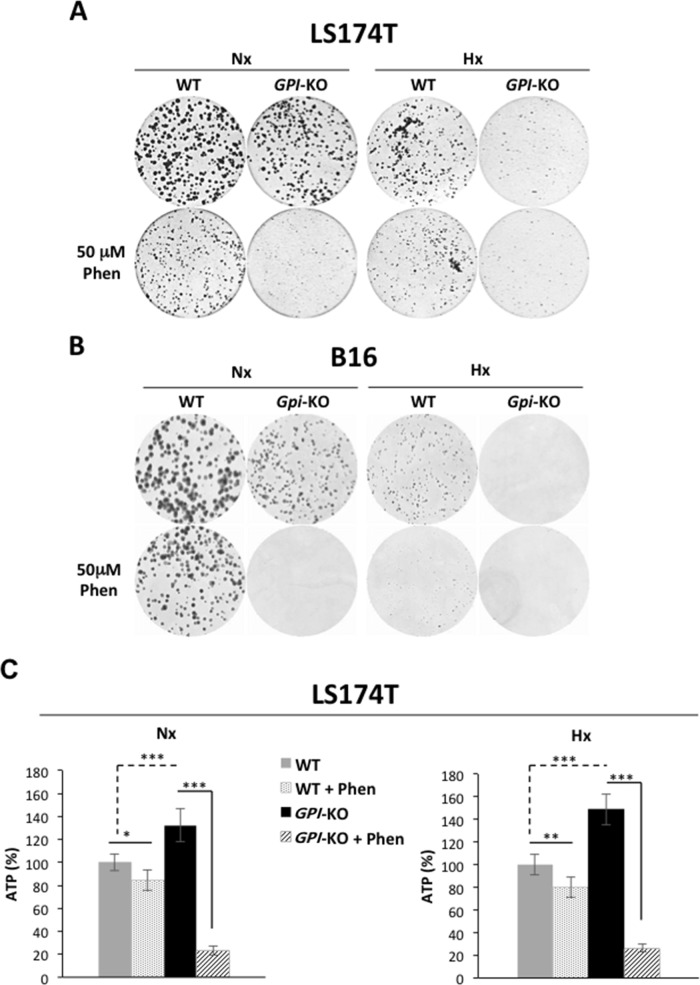
*GPI* disruption sensitizes tumor cells to phenformin both in normoxia and in hypoxia by decreasing cellular ATP levels Clonal growth of LS174T **(A)** and B16 **(B)** WT and *GPI*-KO cells in Nx and Hx, treated or not with 50 μM phenformin (Phen). The results are representative of three independent experiments. **(C)** ATP levels in WT and *GPI*-KO LS174T cells in Nx and 1% Hx in the absence or presence of phenformin (Phen - 50μM) for 24h. Values were standardized to the cell protein content and normalized to initial cellular ATP level (%). The mean ± S.E.M. is representative of two independent experiments carried out in quadruplicate. ^*^ p < 0.05, ^**^ p < 0.001, ^***^ p < 0.0001.

Since *GPI*-null cells rely exclusively on OXPHOS for their energy production, we next compared and explored their clonogenicity/viability in response to the mitochondrial complex I inhibitor, phenformin. As shown in Figure 5A and 5B, the growth of both *GPI-KOs* was restricted in normoxia and more severely in 1% hypoxia. Both LS174T and B16 WT cells showed a slight sensitivity to 50μM phenformin treatment, whereas *GPI*-KO cells were extremely sensitive, both in normoxia and in hypoxia. This loss of viability was shown to be due to the huge drop in ATP levels (Figure 5C) in *GPI*-KO cells treated with phenformin. Note that *GPI*-KO cells, due to their oxidative metabolism, had higher ATP levels with respect to the WT cells, both in normoxia and in hypoxia.

Therefore, we showed that *in vitro*, *GPI*-KO cells (LS174T and B16) have re-routed glucose metabolism *via* the oxidative Pentose Phosphate Pathway, an action that has reduced only by 2-fold the rate of proliferation. However, *GPI*-disruption has suppressed proliferation in hypoxia and rendered these cells extremely sensitive to OXPHOS inhibitors.

### *GPI*-KO increases ROS levels in B16 cells

**Figure 6:**
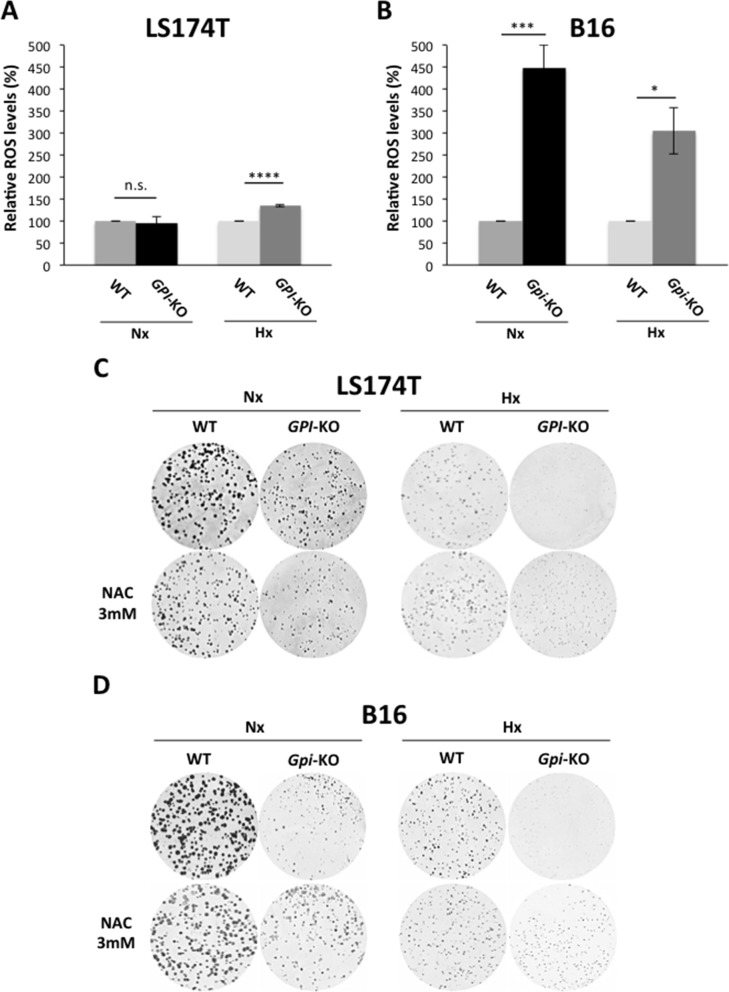
B16 *Gpi*-KO cells display a strong increase in ROS production ROS levels in WT and *GPI*-KO cells in Nx and in Hx at 24h, in LS174T **(A)** and B16 **(B)** standardized to the cell protein content and expressed as percentage of WT Nx, to which a value of 100% was given. The mean ± S.E.M. is representative of five independent experiments carried out in quadruplicate. ^*^ p < 0.0350, ^***^ p < 0.0004, ^****^ p < 0.0001. Clonogenic assay of LS174T **(C)** and B16 **(D)** WT and *GPI*-KO cells in Nx and Hx, in the absence or presence of N-acetyl-L-cysteine (NAC- 3 mM).

Having demonstrated that *GPI*-KO cells have oxidative metabolism and rely on the OXPHOS for their ATP production, we hypothesized that this increase in OXPHOS activity could result in increased reactive oxygen species (ROS) production. Indeed, ROS levels were significantly higher in LS174T cells in hypoxia and in B16 *Gpi*-KO cells both in normoxia and in 1% hypoxia (Figures 6A and 6B). Consequently, treatment with N-acetyl-cysteine (NAC), known to replenish the limiting cysteine precursor of the major cellular antioxidant glutathione, increased the viability of *GPI*-KO cells (Figures 6C and 6D).

### Tumor growth is impacted only moderately by *GPI* disruption

**Figure 7:**
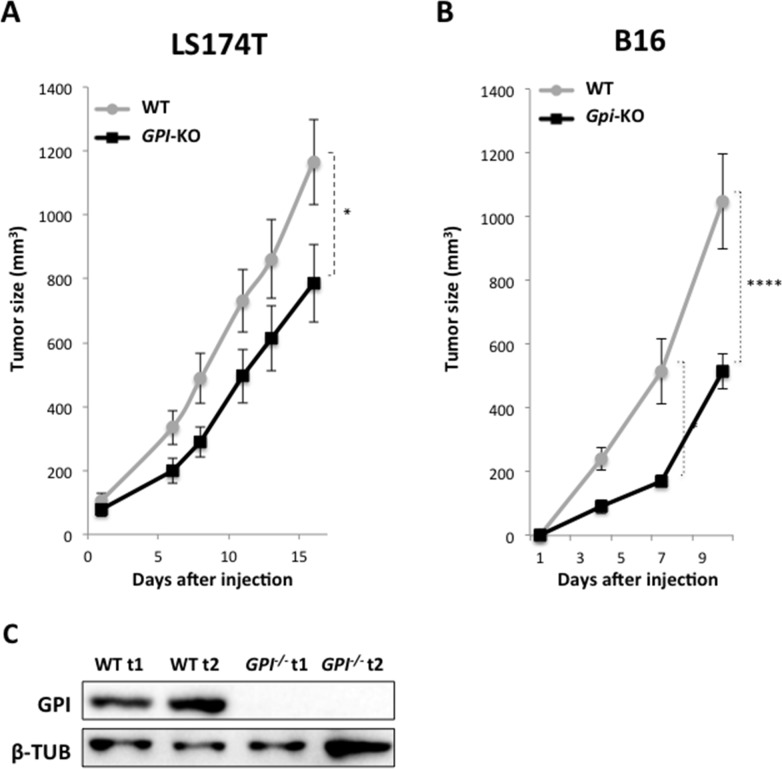
Xenograft tumor growth is minimally impacted by *GPI*-KO in both LS174T and B16 cancer cell lines Tumor volume of WT and *GPI*-KO LS174T cells xenografts **(A)** or mouse melanoma B16 WT and *Gpi*-KO cells **(B)**. 1 × 10^6^ cells were injected in *nude* mice and the experiments were stopped once WT tumors reached 1000mm^3^. ^*^ p < 0.05, ^****^ p < 0.0001. **(C)** Immunoblot analysis of two individual WT and two *GPI*-KO (*GPI^−/−^*t1, *GPI^−/−^*t2) tumors for GPI expression.

To answer the question of whether lack of *GPI* in tumor cells, which caused the shift in their metabolism to OXPHOS *in vitro* will affect their tumorigenicity, we injected subcutaneously 1×10^6^ WT and *GPI*-KO cells, both LS174T and B16, into 8-weeks-old *nude* female mice and monitored the tumor growth rates. The experiment with the LS174T cells was stopped 16 days after the injection, when tumors in WT group reached 1000 mm^3^ (Figure 7A). The onset of tumor appearance was almost indistinguishable between WT and *GPI*-KO cells (Figure 7A), but the kinetics of tumor growth was slightly different. *GPI*-KO tumors showed a slower growth rate and reached approximately 60% of that of the WT tumors by the end of the experiment. The B16 WT tumors showed more aggressive behaviour, and the experiment was stopped after 10 days, when WT tumors reached 1000 mm^3^ (Figure 7B). Similarly to LS174T, B16 *Gpi*-KO tumors had a slower growth rate and reached only about 50% of that of WT tumors, showing that *Gpi* disruption had a modest effect on tumor growth *in vivo*. Tumors were then excised and analyzed for GPI expression by immunobloting (Figure 7C), which confirmed the absence of protein in *GPI*-KO tumors.

## DISCUSSION

### Is glucose-6-phosphate isomerase a moonlight protein?

GPI is an essential glycolytic and gluconeogenic enzyme that catalyzes the reversible isomerization between glucose-6-phosphate and fructose-6-phosphate. This step, found at the intersection of Embden-Meyerhof pathway (glycolysis) and pentose phosphate pathway (PPP), occupies a critical role in glucose metabolic fate. Glucose metabolic flux could be routed through different ‘PPP/glycolysis’ ratios depending on oxygen and glucose concentrations in the microenvironment and the proliferation/differentiation status of the cell. GPI expression, like most glycolytic enzymes, is induced by cMyc [21] and HIF-1 [22] and is increased in many cancers [8, 22]. GPI has also been described as an extracellular cytokine, called autocrine motility factor (AMF), and in conjunction with its receptor gp78/AMFR appears to be involved in several pathways linked to metastasis including glycolysis, matrix remodeling, ER-associated degradation and receptor signaling and endocytosis [23]. However, questions remain about these disparate functions due to lack of firm experimental demonstration and now with the generation of *GPI* genetic disruption it will be important to investigate the alternate functions associated with “secreted” GPI [23]. Multiple functions of GPI are not unique as many ancient metabolic enzymes referred to as moonlight proteins [24, 25] share this multi-functionality that will have to be further investigated and critically assessed by gene disruption.

### Glycolytic “addicted” tumors survive and escape the full ablation of the “Warburg effect”

Most rapidly growing tumors exhibit an increased dependency on glycolysis in comparison to normal tissue. This phenomenon, referred to as glycolytic “addiction”, in place of oxidative phosphorylation for ATP production has long been thought to be a point of cancer vulnerability. Many examples of pharmacological inhibition of members of the upstream (2-DG) [4, 8, 9] or downstream (LDHA) [10–13] glycolytic pathway were shown to be effective in blocking tumor growth but the associated toxicity and low benefit of this approach prevented their development in the clinic [4, 8, 9, 26]. In contrast to these pharmacological studies, our work revealed that genetic abolition of fermentative glycolysis (*GPI*-KO) in the two cell lines LS174T and melanoma B16 does not suppress tumor growth. Tumor cell growth was only reduced by two-fold *in vitro* (normoxia) and *in vivo* (xenografts), whereas in hypoxia (1% O_2_) *in vitro* assays, *GPI*-KO cells survive but stop growing. These findings are consistent with the metabolic plasticity and capacity of metazoans to derive their energy and anabolic precursors from both fermentative and OXPHOS pathways. In contrast to WT cells, the increased dependence on the OXPHOS pathway in *GPI*-KO cells accounts for their extreme sensitivity to respiratory chain inhibitors (oligomycin, phenformin) and oxygen-dependence for growth. Identical findings were recently obtained on the same two cell lines by a double gene disruption of *LDHA*/*LDHB* that fully suppressed fermentative glycolysis with a minimal effect on tumor growth (Maša Ždralević and Jacques Pouysségur, unpublished data). We therefore propose that the previously used glycolysis inhibitors were most effective in blocking tumor growth because of their metabolic dual effects (2-DG) or off-target effects (LDHA inhibitors).

Two recent and interesting studies from Genentech have evaluated tumor growth arrest and metabolism reprogramming by pharmacological blockade of glycolysis at the level of GPI [8] or by more specific inhibitors of LDHA/B [10]. Both studies revealed that most of the tumor cell lines analyzed stopped growing and died with this pharmacological approach. However, some cell lines can reprogram their metabolism and escape the glycolysis blockade in a way that sustains ATP levels, viability and growth as attested by an active mTORC1 (mammalian target of rapamycin complex 1) activity [8, 10]. We cannot exclude that in our genetic approach only a few *GPI*-KO or *LDHA*/*B*-DKO cells were able to escape the blockade of glycolysis and grow because they perform a more successful OXPHOS metabolic reprogramming. Future metabolomics studies will be developed comparing genetic and pharmacologic blockade of glycolysis on these cell lines.

### Lactic acidosis and immunometabolism

Finally, in the context of the tumor microenvironment, lactic acid secretion was recently shown to blunt the immune response in B16 melanoma by inhibiting tumor surveillance by T and NK cells [11, 27]. Similarly, deletion of *LDHA* in myeloid cells promoted accumulation of macrophages with a CD86^high^ and MCP-1^high^ M1-like phenotype that suppressed tumor growth [27]. The question of whether lactate, acidosis or both are involved remains to be addressed. In regard to this question it is important to recall that tumor acidity has been previously reported to be independent of glycolysis [28]. With the melanoma B16 *Gpi*-KO cell line developed here or the *LDHA*/*B*-DKO (unpublished data) in which lactic acidosis is substituted for carbonic acidosis (OXPHOS-dependence), this important question should be simple to address experimentally.

In conclusion, we demonstrated that although complete genetic disruption of the Warburg effect seriously reduced hypoxic *in vitro* cell growth, it is dispensable for *in vivo* tumor growth of aggressive cancers. These findings further illuminate the metabolic plasticity of cancer cells and complicate the clinical anticancer approaches targeting metabolism. As we previously discussed [29, 30], glycolysis inhibition approaches (MCT, LDH, RTKs… inhibitors) will have to be combined with an acute and very short phenformin treatment that we refer to as ‘metabolic knife’ [29]. This anticancer strategy is still awaiting pre-clinical evaluation.

## MATERIALS AND METHODS

### Cell culture and hypoxic exposure conditions

Human colon adenocarcinoma LS174T cells (kindly provided by Dr. Van de Wetering, NL), and mouse B16 F10 (kindly provided by Pr. Marina Kreutz, Regensburg, Germany) were grown, unless otherwise specified, in Dulbecco's modified eagle medium (DMEM, Gibco by Life Technologies Corporation, Paisley, UK) and Roswell Park Memorial Institute (RPMI) medium, supplemented with fetal bovine serum (10%), penicillin (10 U/mL) and streptomycin (10 μg/mL). In normoxic conditions, cells were incubated in a humidified atmosphere with 5% CO_2_/21% O_2_ at 37 °C. In hypoxic conditions the cells were maintained in 1% O_2_ in a sealed anaerobic workstation (*In VIVO*_2_ 400, Ruskinn Technology Ltd, Bridgend, South Wales) where the air was replaced by N_2_ and CO_2_ was maintained at 5%.

### CRISPR/Cas9-mediated knockout of mouse and human *GPI* gene

In order to get *GPI* knockout, human LS174T cells and mouse B16 F10 cells were respectively transfected with CRISPR/Cas9 using pSpCas9(BB)-2A-GFP (PX458). This plasmid was a gift from Feng Zhang (Addgene plasmid #48138) [31] bearing GFP-encoding region designed by J. Durivault (Centre Scientifique de Monaco). The small guide RNA (sgRNA) was designed using the CRISPR Design Tool (http://crispr.mit.edu). Transfections were performed with JetPRIME^®^ (Polyplus transfection, Illkirch, France) and GFP positive cells were detected by cell sorting (FACS). Each clone was analyzed for GPI expression by immunoblot and enzymatic assay. Finally, two independent *GPI* knockout clones for human and mouse cell lines were selected for this study. Since the findings obtained were identical for the two clones, we showed the results of one of the clones for both cell lines for simplicity reasons.

### Immunoblotting

Cells were lysed with SDS buffer and the protein concentration was determined using the BCA assay (Interchim, Montluçon, France). Proteins (40 μg) were separated by SDS-PAGE (10%) and transferred onto a PVDF membrane (Immobilon, Merck Millipore Ltd, Tullagreen, Carrigtwohill, Co. Cork, Ireland). Membranes were blocked in 5% non-fat milk in TN buffer (50 mM Tris-HCl pH 7.4, 150 mM NaCl) and blotted with the antibodies for GPI (Abcam, ab118149), HIF-1α (rabbit anti-human/mouse polyclonal antibody, produced in our laboratory), GLUT1 (Abcam, ab652), TXNIP (MBL, K0204-3) and VDAC1 (Abcam, ab15895). Antibody against ARD1 (rabbit anti-human/mouse ARD1 produced in our laboratory) was used as loading control. Immunoreactive bands were detected with the ECL system (Millipore Corporation, Billerica, MA, USA) after the incubation of membrane with secondary anti-mouse or anti-rabbit antibodies (Promega) and visualized using GeneSys software (Syngene, Cambridge, United Kingdom).

### Preparation of cell extracts for enzymatic assays

Cells (1 × 10^5^) were seeded in 6-well plates and after 24h of incubation in normoxia and hypoxia they were transferred on ice, washed once with 2ml cold PBS, once with 2ml cold dH_2_O, added with 300μl dH_2_O and put at −80 °C for minimum 10 min. Cells were taken out on ice, transferred into eppendorf tubes by scratching, centrifuged at 4 °C (8000 × g for 10 min) and the supernatant corresponding to the cell extract was transferred to new tubes and stored at −80 °C if not analyzed immediately.

### Enzymatic activity assay

Enzymatic assay of glucose-6-phosphate isomerase (GPI) activity in WT and *GPI*-KO cells was performed by continuous spectrophotometric rate determination (Glomax, Promega BioSystems Inc., Sunnyvale, CA, USA). GPI activity was determined by a coupled enzyme assay in which glucose-6-phosphate is converted in 6-phosphogluconate by glucose-6-phosphate dehydrogenase (G-6-PDH), coupled with concomitant β-NADP^+^ reduction to β-NADPH, that can be monitored spectrophotometrically as an increase in absorbance at 340nm, which is the maximum absorption of the reduced cofactor form. This reaction will occur only in presence of GPI in the added cell extract, which will convert fructose-6-phosphate into glucose-6-phosphate. Reaction is performed at pH 7.4 and 25 °C and it is started by adding 40μl of cell extract to reaction mixture containing 42mM glycylglycine buffer, pH 7.4; 3.3mM D-fructose-6-phosphate; 0.67mM β-NADP^+^; 5.0 units of G-6-PDH and 3.3mM MgCl_2_. Results of four independent experiments were reported as mean values of slopes (tangent time 1) corresponding to the initial rate of the reaction, normalized to μg of protein.

### Proliferation and cell viability assay

Cells (5×10^4^ for LS174T and 2×10^4^ for B16) were seeded in 6-well plates in triplicate per cell line and condition. 24h after seeding cells were detached by trypsinization and counted with an automatic cell counter (ADAM-MC™, Digital Bio, NanoEnTek Inc., Seoul, Korea) (day 0). Cell proliferation index was calculated by dividing the cell number obtained for each day by the one obtained 24 hours after seeding.

For determining cell viability, the supernatant taken at indicated time points was collected and cells were washed with PBS, trypsinized, centrifuged (5min, 1000 rpm), added to the supernatant taken previously and resuspended in propidium iodide solution in order to discriminate between live and dead cells. Three independent experiments were performed in duplicate.

### Metabolic flux analysis

Oxygen consumption rates (OCR) and extracellular acidification rates (ECAR) of cells were analyzed by Seahorse XF24 extracellular flux analyzer (Seahorse Bioscience, MA, USA). Cells were seeded on Seahorse plates in order to reach confluency in 24h. One hour prior to measurement, cell media was replaced by the assay medium (without glucose, pyruvate, serum and buffer, D5030, Sigma) and the plates were incubated in a non-CO_2_ incubator at 37 °C. Basal levels of OCR and ECAR were recorded for 24 min, followed by a mitochondrial stress test (1 μM oligomycin, 3 μM FCCP, 1 μM rotenone/1 μM antimycin A). Normalization to protein content was performed after each experiment and data were presented as mpH/min/μg protein for ECAR and as pMolesO_2_/min/μg protein for OCR.

### Extracellular glucose and lactate levels measurement

Cells (1×10^6^) were seeded in 10cm dishes and incubated for 24h and 48h in normoxia and in hypoxia, after which 500μl of supernatant is taken, centrifuged at 8000×g, 4 °C, for 5 minutes and lactate and glucose were analyzed in the same samples with Cobass c701 instrument (Roche Diagnostics, Mannheim, Germany), in collaboration with the Biochemistry laboratory of the Nice University Hospital. The method used is based on enzymatic conversion of lactate into pyruvate by the lactate oxidase, coupled with the colorimetric reaction of hydrogen-peroxide formed in the first reaction with the hydrogen donor, resulting in a formation of colored compound, the intensity of which is measured spectrophotometrically and is directly proportional to the concentration of lactate. Glucose measurements were done by the hexokinase/glucose-6-P dehydrogenase coupled reaction in presence of ATP and NADP^+^. Three independent experiments were performed in duplicate, and the results were normalized to the quantity of the protein and expressed as mM lactate/μg protein.

### MtDNA analysis

Cells (1 × 10^6^) were seeded in 10 cm dishes and after 24h incubation DNA extraction was performed using AllPrep DNA/RNA kit (Qiagen GmbH, Hilden, Germany). 30ng DNA for LS and 0.3ng DNA for B16 cells were used and the ratio of mt/gDNA was determined by quantitative PCR, by amplifying *hNADH*-dehydrogenase and *hLPL* (lipoprotein lipase) for the LS174T cells and mCytB (cytochrome b)/mACT (actin) for B16 cells and calculating the ratio of their expression, using *dd*Ct method. Oligonucleotides for hNADH, hLPL, mCytB and mACT were kindly provided by dr D. Pisani (IBV, Nice, France). The qPCR was performed with Takyon™ Rox SYBR MasterMix dTTP Blue (Eurogentec, Seraing, Belgium) on StepOnePlus Real Time PCR Machine (Applied Biosystems, Life Technologies, Villebon-sur-Yvette, France).

### Clonogenic viability assay

Cells (1×10^3^) were seeded on 60mm plates and incubated for 24h, after which the medium was replaced with DMEM 10% FBS with and without 50 μM phenformin or 3mM NAC. After 8-10 days in normoxia and 12-15 days in hypoxia (1% O_2_), colonies were stained with 5% Giemsa solution (Sigma-Aldrich, Hannover, Germany) for 30 min for colony visualization.

### ATP measurement

Cells (1×10^3^) were plated onto 96-well plate, allowed to adhere and then treated or not with phenformin (50μM) for 24h both in normoxia and in hypoxia. Intracellular ATP levels were measured using a Cell-Titer-Glo Kit (Promega, Madison, WI, USA) according to the manufacturer's instructions. Protein normalization was performed after each experiment and the results were expressed as percentage of initial ATP level.

### ROS levels

Cells (1×10^4^) were seeded onto 96-well-plate (Corning, black plate, clear bottom) in quadruplicate per cell line in normoxic and in hypoxic conditions. Cells were treated with 400μM H_2_O_2_ 1,5h before the measurement as a positive control. Treated and non-treated cells were washed with PBS and incubated with 5μM 2′,7′dichlorofluorescein diacetate (DCF-DA-Sigma-Aldrich) for 30min at 37°C in the dark. DCF was then detected by an Infinite F200 PRO microplate reader (TECAN Trading AG, Switzerland) using excitation and emission wavelengths of 485 and 525 nm, respectively. Four independent experiments were performed and protein normalization was made after each experiment.

### Tumour xenografts

To generate tumors in mice, 1 × 10^6^ cells were suspended in 300 μL of serum-free DMEM supplemented with insulin-transferrin-selenium (Gibco, Invitrogen Corporation, Paisley, Scotland, UK) and injected subcutaneously into the back (LS174 cells) or the flank (B16 cells) of 8-week-old *nude* female mice (Harlan). Tumor dimensions were measured every 3–6 days using a caliper and the volume was determined by using the formula: (4π/3) × L/2 × W/2 × H/2 (L: length, W: width, and H: height). Once the tumor volume reached 1000mm^3^, mice were euthanized and the tumors were collected. All procedures were approved by i) the Institutional Animal Care and Use Committee of the University of Nice-Sophia Antipolis (CIEPAL-azur agreement NCE/165) and ii) the animal experimentation protocol of the local animal care committee (Veterinary service and direction of sanitary and social action of Monaco, Dr H. Raps).

### Statistical analysis

Results were expressed as mean ± SEM and statistical analysis was performed using ANOVA followed by non-parametric tests, using GraphPad Prism 5 Software. The differences between the groups were considered significant when p < 0.05.

## Abbreviations

GPI: glucose-6-phosphate isomerase
PPP: pentose phosphate pathway
OXPHOS: oxidative phosphorylation
NAC: N-acetyl-L-cysteine
Nx: normoxia
Hx: hypoxia
G6P: glucose-6-phosphate
TXNIP: thioredoxin-interacting protein
OCR: oxygen consumption rate
ECAR: extracellular acidification rate
ROS: reactive oxygen species
